# Etiology, diagnosis, and management of chronic pancreatitis associated primary complications: a narrative review

**DOI:** 10.3389/fmed.2026.1822750

**Published:** 2026-07-09

**Authors:** Liqi Sun, Mengruo Jiang, Xiaoyun Wang, Yuwei Sun, Yatao Tu, Zhaoshen Li, Lisi Peng, Haojie Huang

**Affiliations:** 1 Department of Gastroenterology, National Clinical Research Center for Digestive Diseases, Changhai Hospital, Navy Medical University, Shanghai, China; 2 Department of Gastroenterology, 72nd Group Army Hospital, Huzhou University, Huzhou, Zhejiang, China; 3 National Key Laboratory of Immunity and Inflammation, Naval Medical University, Shanghai, China

**Keywords:** chronic pancreatitis, diagnosis, etiology, management, primary complications

## Abstract

Chronic pancreatitis is a progressive inflammatory disease, whose hallmark pathological feature is an irreversible fibrosis process. This process further leads to irreversible structural damage, pancreatic functional decline, and a variety of severe complications. Common complications mainly include pancreatic exocrine insufficiency, endocrine insufficiency, metabolic bone disease, pain, pancreatic duct stones, obstruction of common bile duct and duodenum, pancreatic pseudocysts, and vascular complications. This review systematically sorts out and integrates the etiology, diagnostic criteria, and management of various complications. Moreover, active intervention for these complications through secondary prevention and standardized management is crucial for improving patients’ clinical prognosis. Ultimately, this review establishes a comprehensive “Etiology-Diagnosis- Management “clinical reference system for the primary complications of chronic pancreatitis, providing gastroenterologists with a quick reference for the standardized diagnosis and management of chronic pancreatitis-related complications.

## Introduction

Chronic pancreatitis (CP) is a progressive fibro-inflammatory condition initiated by abnormal responses to pancreatic injury or stress in susceptible individuals. This leads to irreversible structural damage (atrophy, fibrosis, duct distortion/strictures, calcifications), functional decline (exocrine and endocrine insufficiency), and clinical manifestations like pain syndromes and dysplasia in its advanced stages. While often multifactorial, its exact pathophysiology remains incompletely elucidated (1). Globally, CP occurs at an estimated rate of approximately ten cases per 100,000 person-years (2). Men experience this condition twice as frequently as women, and recent epidemiological data (past 5 years) suggest an increasing incidence trend (3).

Significant patient heterogeneity contributes to an unpredictable disease course in CP. Affected individuals are at risk for multiple complications. Mitigating these complications through secondary prevention and management is fundamental to improving clinical outcomes. However, existing literature reviews still lack a summary of primary complications associated with CP. Therefore, in this review, we aim to describe the etiology, diagnosis, and management of these CP associated primary complications to provide quick references for gastroenterologists (Table 1).

**Table 1:** The primary complications related to CP: incidence, major diagnostic methods, and main treatment approaches.

Type of complications	Incidence	Major diagnostic method	Main treatment approach	Important limitations/controversies
Exocrine insufficiency	20% at 5 years after onset to 70% by 20 years	Fecal elastase-1 (<200 μg/g abnormal; <100 μg/g consistent with PEI)	PERT: start ≥40,000 USP lipase/meal, then individualize by fat intake	FE-1 insensitive for mild PEI; optimal dosing (fixed vs. fat-adjusted) debated
Endocrine insufficiency (type 3c diabetes)	4–5% of all diabetes	Ewald and Bretzel criteria: (1) PEI, (2) abnormal imaging, (3) no type 1 autoantibodies	Metformin or insulin; avoid incretins	Criteria overlap with type 2 diabetes; no standardized algorithm
Metabolic bone disease	17% ~ 23% for osteoporosis and 39% for osteopenia	DEXA (T-score ≤ − 2.5 = osteoporosis; −1 to −2.5 = osteopenia)	Vitamin D (800–2000 IU/d) + calcium; bisphosphonates if osteoporosis	Screening intervals not standardized; role of vitamin K₂ unclear
Pain	84% ~ 90%	Clinical: structural changes on imaging + temporal association	Stepwise: lifestyle → analgesics → endoscopy/surgery	Early surgery vs. endoscopy-first remains debated
Pancreatic duct stone	Increases with time to reach 50 and 100%, at 5 and 14 years after the onset of the disease	CT/MRCP: size, location, number, radiopacity	ESWL + ERCP or pancreatoscopy-guided lithotripsy	No RCT comparing ESWL vs. primary pancreatoscopy
Obstruction of common bile duct and duodenum	<1% ~ 8%	Imaging (CT/MRCP) or endoscopy	Endoscopic stenting (biliary/duodenal) or surgery (hepaticojejunostomy)	Distinguishing groove pancreatitis from cancer is difficult
Pseudocysts	11.41% ~ 14.7%	Imaging: defined wall, no antecedent acute pancreatitis	Drainage if symptomatic (>4–6 weeks, >5 cm, or complications)	Plastic vs. metal stent; LAMS for solid debris vs. liquid
Vascular complications	12.2%	Doppler ultrasound, CTA/MRA, or DSA	Variceal bleed: endotherapy; asymptomatic thrombosis: observation ± anticoagulation	Anticoagulation role not evidence-based; spontaneous recanalization in ~30%

PEI, pancreatic exocrine insufficiency; PERT, pancreatic enzyme replacement therapy; DEXA, dual-energy X-ray absorptiometry; ESWL, extracorporeal shock wave lithotripsy; ERCP, endoscopic retrograde cholangiopancreatography; CBD, common bile duct; CTA, computed tomography angiography; MRA, magnetic resonance angiography; DSA, digital subtraction angiography; LAMS, lumen-apposing metal stent.

Given the nature of this review as a narrative synthesis rather than a formal systematic review, we did not follow PRISMA guidelines. However, to ensure comprehensive coverage of the literature, we conducted a structured search of PubMed, Web of Science, and Cochrane Library databases from inception to October 2025 using the following keywords: “chronic pancreatitis” combined with “exocrine insufficiency,” “endocrine insufficiency/type 3c diabetes,” “metabolic bone disease/osteoporosis,” “pain,” “pancreatic duct stone,” “bile duct obstruction,” “duodenal obstruction,” “pseudocyst,” and “vascular complications.” Articles were included if they were published in English, involved human subjects, and provided original data, consensus guidelines, or systematic reviews on the diagnosis and management of CP-related complications. Priority was given to high-quality randomized controlled trials, meta-analyses, and major society guidelines. Reference lists of retrieved articles were hand-searched for additional relevant studies.

## Exocrine insufficiency

Pancreatic exocrine insufficiency (PEI) arises from damage to or loss of acinar cells or obstruction of pancreatic outflow (e.g., due to duct strictures or stones) (4, 5). Clinically, it is characterized by a reduction in pancreatic enzyme secretion and/or intraluminal activity below the threshold required for normal nutrient digestion. This deficiency leads to malabsorption, potentially causing intestinal symptoms such as steatorrhea, diarrhea, bloating, flatulence, and nutritional deficiencies (6).

### Etiology

In patients with CP, the prevalence of PEI shows a strong association with disease duration and smoking history. Prevalence rises from approximately 20% at 5 years after onset to roughly 70% by 20 years (1, 7).

### Diagnosis

Pancreatic function tests provide objective, quantitative assessment of exocrine pancreatic synthesis and secretion. While direct tests, measuring secretions in the duodenum, offer the highest accuracy, they are invasive, time-consuming, and burdensome for patients, limiting their use to specialized centers (typically via endoscopic methods) (4, 8). Modern direct techniques involve pancreatic stimulation followed by 30–60 min aspiration of secretions for analysis of bicarbonate and digestive enzymes (9). These tests are primarily employed for diagnosing early CP rather than established PEI.

Indirect tests, notably fecal human elastase-1 (FE-1), are preferred clinically due to simplicity, noninvasiveness, and cost-effectiveness (4, 10). Elastase-1 survives intestinal transit well, making stool levels a reliable proxy for pancreatic enzyme output. Under controlled conditions, FE-1 distinguishes between normal function and moderate or severe PEI. Interpretation uses established cutoffs: <200 μg/g stool: Abnormal; <100 μg/g stool: Consistent with PEI; <50 μg/g stool: (Some suggest most reliable for severe PEI) (11, 12). However, FE-1 testing has limitations: limited sensitivity for mild PEI and optimal performance only with formed or semi-formed stool. Indeterminate results warrant retesting, particularly in patients with pancreatic disease and high clinical suspicion of PEI (11). Moreover, the pancreatic enzyme replacement therapy (PERT) does alter the results of FE-1 (13).

Fecal fat testing is now rarely indicated. When performed, it requires a high-fat diet and quantitative analysis is generally impractical for routine use. Its primary role is reserved for cases where clinical features are inconclusive (to confirm steatorrhea) or when assessing an inadequate response to PERT (14). Notably, response to a therapeutic trial of PERT is unreliable for diagnosing PEI. While patients with nonspecific symptoms like bloating, excess gas, or stool abnormalities (foul-smelling, floating) may report improvement on PERT, these symptoms lack specificity. Symptomatic changes could reflect placebo effects or mask other conditions (e.g., celiac disease), potentially delaying correct diagnosis. Although cross-sectional imaging is crucial for diagnosing the underlying causes of pancreatic disease, it is unreliable for directly identifying PEI itself (15).

### Treatment

The goals of PEI in CP patient treatment are the steatorrhea and associated gastrointestinal symptom improvements, a gain of weight, muscle mass, and muscle function; and improvement in fat-soluble vitamin levels.

Before all the treatment measures, dietary modifications include a low-moderate fat diet with frequent smaller meals and avoiding very-low-fat diets are required.

Initiation of PERT is recommended as the fundamental treatment for PEI (4). PERT should be taken during the meal, with the initial treatment of at least 40,000 USP units of lipase during each meal in adults and one-half of that with snacks. However, even at high doses, PERT formulations are not as efficient as normal pancreatic secretion and do not correct steatorrhea entirely. Therefore, the subsequent dosage can be adjusted based on the meal size, fat content and symptom improvement (4, 16, 17). This recommendation is based on a 2009 systematic review and a 2017 meta-analysis; more recent individual studies are limited.

During the disease course of CP patients with PEI, routine monitoring fat-soluble vitamin levels, body mass index, and bone mineral density (BMD) should be obtained. Timely supplementation of vitamins and calcium may prevent the PEI associated complications.

### Prevention

Prevention of PEI progression relies on smoking cessation and early PERT initiation when mild insufficiency is detected (FE-1100–200 μg/g).

## Endocrine insufficiency

In CP patients, endocrine insufficiency predominantly manifests as diabetes mellitus. Throughout the disease progression, all pancreatic islet cell types are compromised, leading to deficiencies in insulin, glucagon, and pancreatic polypeptide. This hormonal dysregulation causes significant and rapid fluctuations in blood glucose levels, characteristic of brittle diabetes (type 3c diabetes) (18, 19). Previous studies indicate that compared to type 2 diabetes patients, those with type 3c diabetes exhibit poorer glycaemic control, higher rates of diabetes-related complications, and greater requirements for antidiabetic medications (20, 21).

### Etiology

Among patients with CP, new-onset diabetes incidence is estimated to reach up to 50% within 10 years of diagnosis and is associated with disease duration, smoking, pancreatic calcifications, and pancreatic surgery (22). Among all the patients with diabetes, the true prevalence of type 3c diabetes probably ranges from 1 to 9% of patients with diabetes, and 4–5% might be a reasonable working estimate (18).

### Diagnosis

Ewald and Bretzel established major diagnostic criteria for CP-associated type 3c diabetes (all mandatory): (1) Exocrine pancreatic insufficiency (confirmed by monoclonal fecal elastase-1 [FE-1] testing or direct function tests), (2) Characteristic pancreatic abnormalities on imaging (endoscopic ultrasound, MRI, or CT), and (3) Absence of type 1 diabetes-associated autoimmune markers. Minor criteria include impaired *β*-cell function (measured via HOMA-β, C-peptide, or glucose levels), absence of insulin resistance (defined by HOMA-IR), impaired incretin secretion (GLP-1 and/or pancreatic polypeptide), and low serum lipid-soluble vitamins (A, D, E, K) (23).

However, these criteria face significant limitations: overlap with features of long-standing type 1 or 2 diabetes, lack of standardization in assessing *β*-cell function and insulin resistance, scarcity of high-quality data on glucose homeostasis in type 3c diabetes, the frequent presence of coexistent insulin resistance in patients, and variable availability of required radiological and laboratory resources. Consequently, differentiating diabetes types clinically remains complex, and the term “new-onset diabetes after CP” is often used synonymously with type 3c diabetes (24).

### Treatment

First-line therapy for CP-related type 3c diabetes involves metformin or insulin, tailored to the patient’s presentation. Cohort data indicate insulin is used in at least half of patients (25), effectively addressing the core insulin deficiency and recommended by consensus guidelines, especially in advanced disease (26). However, insulin carries a hypoglycemia risk, potentially heightened by enhanced peripheral insulin sensitivity in CP patients (27). Conversely, metformin alone may be suitable early in the disease course, particularly for mild hyperglycaemia (HbA1c < 8% or <64 mmol/mol). Metformin offers a theoretical advantage over insulin regarding potential protection against pancreatic ductal adenocarcinoma, though this benefit remains unconfirmed in CP physiology, characterized by insulin deficiency rather than the hyperinsulinemia of type 2 diabetes (28).

The role of other antidiabetic agents is less defined. Incretin-based therapies (GLP-1 analogues, DPP-4 inhibitors) are typically avoided due to potential associations with acute pancreatitis and pancreatic ductal adenocarcinoma (29), based on observational data and mechanistic concerns. Thiazolidinediones, which enhance insulin sensitivity, increase fracture risk and are poorly suited for CP patients already predisposed to this complication (30).

### Prevention

To potentially delay the onset of type 3c diabetes, smoking cessation and maintenance of pancreatic parenchyma through early ductal decompression may be beneficial, though prospective trials are lacking.

## Metabolic bone disease

Metabolic bone diseases (MBDs) constitute a diverse group of systemic skeletal disorders, mainly including osteoporosis, fibrous dysplasia, and osteogenesis imperfecta (31). CP disrupts both the exocrine and endocrine functions of the pancreas. Such disruptions often lead to nutritional and metabolic imbalances, as pancreatic enzyme secretion decreases and insulin production may become compromised. Consequently, MBDs become one of the most common complications of CP, especially the osteoporosis.

Beyond nutritional deficiencies, the gut microbiota dysbiosis, ferroptosis and inflammation contribute significantly to the pathogenesis of CP-related MBDs. These mechanistic insights are enabling more effective therapeutic strategies for CP-related MBD (32).

### Etiology

A meta-analysis indicated that approximately two-thirds of CP patients have bone disorders, with osteoporosis at 23.4% and osteopenia at 39.8% (33). Supporting this, a recent screening study of 282 CP patients found 56.0% had bone disorders, comprising osteoporosis in 17.0% and osteopenia in 39.0% (34).

The risk of fractures in CP patients is also higher than healthy group. Bang et al. (35) reported that 21.7% of CP patients had experienced fractures, representing a 1.7-fold higher risk compared to age- and gender-matched healthy controls. A larger-scale study involving 3,257 male CP patients and 450,655 healthy individuals further confirmed this increased risk, demonstrating substantially higher rates of overall fractures (2.35-fold), vertebral fractures (2.11-fold), and hip fractures (3.49-fold) among CP patients (36).

### Diagnosis

The onset of CP-associated MBDs often treads a covert path, making their detection and diagnosis challenging until they reach an advanced stage.

The BMD measured by dual-energy X-ray absorptiometry is gold standard to diagnosis osteoporosis and osteopenia. Osteoporosis is defined by BMD at the lumbar spine, total hip or femoral neck of 2.5 standard deviations (T-score) or more below the population mean for young healthy adults (peak bone mass). If T-score lies between −1 – −2.5, the diagnosis is osteopenia (37). The secondary osteoporosis caused by other underlying diseases (congestive cardiac failure, chronic obstructive lung disease, chronic renal insufficiency, etc.) should be excluded.

Bone turnover levels can be assessed using specific biochemical markers. The recommended markers are serum procollagen type I amino-terminal propeptide, reflecting bone formation, and serum carboxy-terminal collagen crosslinks, indicating bone resorption (38). While not diagnostic tools, these markers are valuable for monitoring treatment response and identifying therapeutic failure (39). Importantly, greater reductions in bone turnover markers during anti-resorptive therapy correlate with larger decreases in fracture risk.

### Treatment

Supplementation of key nutrients, maintaining appropriate physical activity, and optimizing CP management constitute the primary strategies for preventing and treating CP-related MBDs. For treating osteoporosis, conventional pharmacological options include: (1) Anti-resorptive agents: such as bisphosphonates, selective estrogen receptor modulators, RANK ligand monoclonal antibodies (e.g., denosumab), and calcitonin. (2) Bone-forming agents: such as parathyroid hormone analogs (e.g., teriparatide) and anti-sclerostin monoclonal antibodies (e.g., romosozumab). (3) Other active agents: including active vitamin D analogs and vitamin K₂ (menaquinone). The probiotics and prebiotics supplementation can be helpful, but needs further large-scale study to verify (40, 41).

### Prevention

Prevention includes routine DEXA screening every 2–3 years in all CP patients, along with vitamin D (800–2000 IU/day) and calcium supplementation, even before BMD declines.

## Pain

CP is characterized primarily by pain, which exhibits various patterns. These include continuous pain (with or without superimposed attacks) and intermittent pain, characterized by pain-free periods between attacks (42, 43).

The pathophysiology of this pain involves multiple mechanisms. Increased ductal and interstitial pressures, nervous system sensitization, generalized hyperalgesia, pancreatic neuroplasticity, and elevated cholecystokinin concentrations all contribute to the painful experience in CP (44). Consequently, effective pain management remains a significant challenge.

### Etiology

About 84–90% of patients with CP experience pain at some point during their clinical course (42, 45). Moreover, patient-reported pain intensity and pattern correlate poorly with structural pancreatic changes and frequently evolve over time (46).

### Diagnosis and assessment

Diagnosing pain associated with chronic pancreatitis (PACP) is relatively straightforward. The primary criteria rely on documented pancreatic structural changes characteristic of CP and the temporal association between pain onset and the disease course.

Different from diagnosis, a comprehensive and accurate assessment can make clinical pain management easier, more accurately record treatment responses and outcomes, and improve the pain experience of CP patients. However, the assessment tools may not meet the demand of clinical practice. In the pain assessment tools for CP patients, only the Comprehensive Pain Assessment Tool for Chronic Pancreatitis has been fully validated (47). Moreover, only the brief pain inventory and McGill pain questionnaire, two multidimensional pain assessment tools, have been clinically validated after being translated into multiple languages (48).

### Treatment

The patient self-reported nature and lack of precise assessment tool for PACP making the accurate treatment decision relatively difficult. The management of PACP requires a multidisciplinary approach. Both domestic and international guidelines advocate a stepwise strategy, progressively escalating treatment intensity to address PACP (49, 50).

First, all patients are advised to quit smoking and abstain from alcohol, and it is also recommended to improve their dietary structure with small, frequent meals and a low-fat diet (50).

Second, pharmacological management typically adheres to the WHO analgesic ladder, progressing sequentially from non-opioids (NSAIDs or acetaminophen) to weak opioids, and finally to strong opioids if required (51). Adjuvant analgesics, such as tricyclic antidepressants, gabapentinoids, selective serotonin inhibitors, and esketamine, have proven to be efficacious in the treatment of chronic pain (not specifically chronic pancreatitis) in multiple placebo-controlled trials (52). In a single randomized controlled trial (RCT), pregabalin showed the superiority over placebo in the PACP treatment (53). The antioxidant therapy or PERT are not essential for PACP management (54, 55).

Third, for the 30–60% of CP patients whose pain remains refractory to medical therapy, invasive interventions—either endoscopic or surgical—become necessary. Current guidelines, such as those from the American Society for Gastrointestinal Endoscopy, recommend surgery as the preferred approach for patients with painful CP and an obstructed main pancreatic duct who are surgical candidates (56). This recommendation is supported by RCTs demonstrating surgical management’s superiority over endoscopy across multiple outcomes: sustained pain relief (partial or complete), technical success (achieving ductal decompression), and physical quality-of-life improvements (57–60). Additionally, surgery proves more cost-effective than endoscopy, yielding greater reduction in pain scores and gains in quality-adjusted life-years per unit cost (61). Despite the recommendation for early surgical evaluation in patients with PACP, the less-invasive endoscopic approach is often preferred in clinical practice before considering surgery.

For endoscopic therapy, endoscopic retrograde pancreatography is primarily suitable for obstruction in the head or neck of the pancreatic duct with endoscopic stenting. When symptoms are improved after insertion of a single plastic stent, long-term (12 months), uninterrupted stenting is required to accomplish remodeling of the pancreatic duct (62). The pancreatic duct stone should be removed at the same time (discussed in the next section).

For patients with PACP refractory to medical therapy, endoscopic ultrasound-guided celiac plexus block represents a potential intervention. Although its therapeutic efficacy and duration are less optimal than for pancreatic cancer-related pain, approximately 50% of PACP patients achieve meaningful symptom relief with this technique, alongside significant reductions in opioid analgesic requirements (63, 64), based on two moderate-sized cohort studies.

The tailored surgery—entailing the least extensive resection based on pancreatic morphology—is the recommended surgical approach. This strategy directly addresses pathological changes in pancreatic structure (65). Surgical therapy can be divided into drainage procedures (e.g., lateral pancreatojejunostomy), resection procedures (e.g., partial pancreatoduodenectomy, distal pancreatectomy, or total pancreatectomy), and a combination of both (e.g., duodenum-preserving pancreatic head resection, including Frey and Berne procedures). However, different surgical approaches exhibit similar clinical outcomes (66). Therefore, in clinical practice, type of procedure is also affected by a surgeon’s preference and local expertise.

### Prevention

Smoking cessation is the single most effective preventive measure to reduce pain progression; patients should be counseled and offered cessation support at every visit.

## Pancreatic duct stone

Pancreatic stones (PS), a direct sequela of CP, can cause pancreatic duct hypertension and tissue ischemia, leading to CP-associated pain and pancreatic atrophy. Therefore, PS is responsible for the dominant symptoms and complications in patients with CP (67).

### Etiology

PS can occur in about 50% of patients. Their prevalence increases with time to reach 50 and 100%, at 5 and 14 years after the onset of the disease (68).

### Diagnosis

Based on the imaging findings, the diagnosis of PS is not difficult. Moreover, the characteristics of PS should be clarified to guide the treatment decision. PS can be classified based on their type, numbers, and location: radio opaque, radiolucent, or mixed; single or multiple; located in the main pancreatic duct, side branches, or in the pancreatic parenchyma; and located in the head, body, or tail region (69).

### Treatment

Endoscopic modalities include endoscopic retrograde cholangiopancreatography (ERCP) with stone extraction, ERCP with intraductal lithotripsy, and extracorporeal shock.

wave lithotripsy (ESWL). Endoscopic lithotripsy methods include mechanical lithotripsy, pancreatoscopy-guided electrohydraulic lithotripsy, and pancreatoscopy-guided laser lithotripsy (56). Surgical modalities include both drainage and resection procedures (2, 65).

Removal of small main pancreatic duct stones (<5 mm) can be attempted endoscopically using extraction balloons or baskets following ERCP-guided pancreatic sphincterotomy. If distal strictures are present, endoscopic dilation (balloon or passage) may be necessary prior to stone removal (70). Risk factors for ERCP failure alone include stones >10 mm, diffuse stone distribution, impacted stones, and downstream strictures (56).

ESWL is indicated for symptomatic chronic calcific pancreatitis patients with large ductal calculi (>5 mm) and ductal dilation, particularly targeting stones in the head or body. ESWL is contraindicated in cases of extensive stone burden, multifocal pancreatic duct strictures, or isolated tail calculi due to splenic injury risk (71). ESWL may be performed alone or followed by ERCP. While spontaneous stone clearance after ESWL allows standalone treatment in some centers, most combine it with subsequent endoscopic procedures (ERCP, sphincterotomy, duct clearance) either immediately or later (72).

Pancreatoscopy-guided lithotripsy (electrohydraulic or laser) has emerged as a newer technique. Evidence suggests it achieves comparable ductal clearance and complication rates to ESWL (73, 74), although most studies were retrospective. In high-volume centers, pancreatoscopy-guided lithotripsy may be preferred over ESWL for pancreatic stone treatment due to potentially requiring fewer sessions.

The role of surgery has been discussed in the previous section.

### Prevention

Early endoscopic or surgical decompression of the main pancreatic duct in patients with recurrent acute pancreatitis or early CP may reduce the risk of stone formation and ductal hypertension.

## Obstruction of common bile duct and duodenum

Chronic inflammation in the pancreatic head, particularly in groove pancreatitis (a specific form affecting the groove between the pancreatic head, duodenum, and common bile duct) and pseudocysts, can compress or involve adjacent structures. Duodenal obstruction may cause gastric outlet obstruction, while common bile duct obstruction can lead to abdominal pain, jaundice, or cholangitis (75).

### Etiology

Determining the exact prevalence of common bile duct (CBD) or duodenal obstruction caused by groove pancreatitis is challenging. Based on surgical series, groove pancreatitis itself accounts for 3–24% of CP cases requiring surgery (76). Among these groove pancreatitis patients, approximately one-third develop vomiting or jaundice-symptoms indicative of CBD or duodenal obstruction. Consequently, the prevalence of these specific obstructive complications is estimated to be less than 1–8% across all CP patients (76).

### Diagnosis

The accurate diagnosis of duodenal or common bile duct obstruction is not difficult by imaging or endoscopic modalities. However, the definite diagnosis of groove pancreatitis is not easy. Importantly, the groove pancreatitis should be distinguished from pancreatic cancer. Patients with groove pancreatitis seemed to be more likely to be male, younger, smokers, heavy drinkers, without jaundice, with pain, with cystic changes in the duodenal wall, and with inflammatory thickening of the second part of the duodenum compared to those with pancreatic cancer (76, 77). EUS guided fine needle tissue acquisition can be helpful in differentiation diagnosis, but the procedure is difficult to perform for lesions in that location of the pancreas (3, 78).

### Treatment

Management begins conservatively for obstructions potentially stemming from pancreatic head edema or pseudocyst compression of the common bile duct or duodenum. If the obstruction persists for 4 weeks or longer, endoscopic therapy is advised: utilizing either multiple parallel plastic stents or a fully covered metal stent for common bile duct involvement (72). For persistent duodenal obstruction, EUS guided gastroenterostomy can be an option (79, 80).

Surgical alternatives, like hepaticojejunostomy, are considered when endoscopic approaches fail or are impractical. Should resection of the pancreatic head be concurrently indicated, procedures include duodenum-preserving pancreatic head resection or pancreatoduodenectomy (81).

### Prevention

Prevention of biliary or duodenal obstruction relies on early control of inflammation in the pancreatic head, particularly in patients with groove pancreatitis. Smoking and alcohol cessation may reduce the risk of progressive inflammatory enlargement of the pancreatic head.

## Pseudocysts

Pancreatic pseudocysts are formed when pancreatic ducts rupture, leading to accumulation of pancreatic fluid collection and necrotic material. Pseudocysts can compress surrounding organs and cause corresponding symptoms. Moreover, complications such as bleeding and infection can occur within the cyst (82). Therefore, the treatment of pseudocyst is of great significance but remains a challenge.

### Etiology

In a cohort of 1998 CP patients, the pancreatic pseudocysts were detected in 228 (11.41%) patients (83). In another cohort of 1,633 idiopathic CP patients, the pancreatic pseudocysts were detected in 240 (14.7%) patients (84).

### Diagnosis

Chronic pseudocysts are diagnosed based on: a definable wall on imaging, no history of preceding acute pancreatitis, and discovery at the time of or following a diagnosis of CP (85).

### Treatment

Spontaneous resolution of pseudocyst is rare (0–27%), mainly in small (<4 cm) and localized cases (86). Therefore, the small asymptomatic cysts can be monitored.

Drainage is indicated for symptomatic pseudocysts manifesting as abdominal pain, fever, infection, or upper digestive tract obstruction. Intervention is also warranted for complications including vascular or biliary compression, pancreato-pleural fistula, cysts exceeding 5 cm without size reduction after 6 weeks, and cases causing significant duct distortion (56).

Endoscopic drainage is preferred for uncomplicated CP pseudocysts accessible via endoscopy rather than percutaneous or surgical methods (87). Among endoscopic procedures, endoscopic transpapillary drainage is recommended for small pseudocysts (<5 cm) communicating with the main pancreatic duct in the head or body of the pancreas. Transmural drainage (EUS-guided) is the preferred approach for other pseudocysts (88).

For pseudocysts containing only liquid, double-pigtail plastic stents are typically favored due to the lower cost, and the stents should be removed at least 6 weeks after pseudocyst regression (89). For patients with main pancreatic duct disconnection, the long-term placement of transmural double-pigtail plastic stent is indicated, based on a single-center observational study (90). For pseudocysts containing solid necrotic, the FCSEMS such as LAMS may be considered due to the large diameter for possible endoscopic transgastric necrosectomy (91).

### Prevention

Prevention of pseudocyst formation primarily focuses on control of acute inflammatory episodes and ductal decompression; no specific therapy is available once CP is established.

## Vascular complications

Patients with CP can experience various vascular complications, broadly categorized as arterial or venous. Venous complications primarily involve splanchnic venous thrombosis (affecting the splenic, portal, or mesenteric veins, either singly or in combination), with inferior vena cava and renal vein thrombosis occurring rarely (92). Arterial complications include pseudoaneurysm formation, percutaneous drain-related arterial bleeding, arterial thrombosis, and rarely, aortic pseudoaneurysm (93).

Venous thrombosis may be either asymptomatic and detected incidentally or present as various clinical manifestations such as gastro-esophageal varices. These complications can be life-threatening due to massive bleeding. Therefore, early recognition and prompt intervention are crucial for achieving favorable outcomes.

### Etiology

Vascular complications in CP patients show significant long-term risk. One study of 394 patients found the cumulative incidence of vascular events rising from 3.2% at 5 years to 24.5% at 15 years (94). Regarding venous complications: splanchnic vein thrombosis has a pooled prevalence of 11.6% (based on a meta-analysis of 44 studies), though individual studies report prevalence rates ranging widely from 3 to 41% (4, 95). For arterial complications, the pooled incidence rate of arterial pseudoaneurysm was notably lower at 0.03% (meta-analysis of 29 studies) (96).

### Diagnosis

The diagnosis is established by imaging modalities such as esophagogastroscopy (EGD) ultrasound doppler, EUS, multidetector CT angiography, MRA or conventional angiography [digital subtraction angiography (DSA)]. Most of these imaging tests are done for non-bleed related indications.

Among these modalities, EUS and DSA are both diagnostic as well as therapeutic, as definitive obliteration can be done in the same setting for arterial bleeding (97).

### Treatment

For venous bleeding, acute management of GI bleed includes resuscitation by intravenous crystalloids and airway protection, followed by EGD and endotherapy for the variceal and non-variceal sources of bleed. In case of variceal bleed, the endotherapy depends on the source of bleed and involves variceal band ligation or sclerotherapy for esophageal varices or cyanoacrylate glue injection into the gastric varices (98).

Optimal management of venous thrombosis in CP patients remains debated. Given the frequent asymptomatic presentation and spontaneous recanalization (occurring in up to 30% of cases), a universal approach is unclear (99). However, all affected patients, especially those with splenic vein thrombosis, should undergo EGD to screen for varices. Evidence supporting anticoagulation—including newer oral agents—in CP-related thrombosis is scarce (100). No randomized data exist; current practice relies on case series and expert opinion. Splenectomy or splenic artery embolization can be considered in some cases with recurrent variceal bleed.

Arterial bleeding is relatively uncommon compared to venous bleeding in CP patients. Management is highly individualized and beyond the scope of this review.

### Prevention

Smoking cessation and optimal control of inflammation may reduce the risk of vascular complications, although direct evidence is limited.

## Controversies and unresolved issues

Despite the comprehensive framework provided above, several areas of clinical practice in chronic pancreatitis complications remain controversial or lack high-quality evidence.

1. Endoscopic versus surgical drainage for painful obstructive chronic pancreatitis. Major society guidelines (e.g., ASGE, ESGE) recommend surgery as the preferred approach for eligible candidates with a dilated main pancreatic duct, based on RCTs showing superior long-term pain relief and cost-effectiveness^[ref]^. However, in real-world practice, endoscopy is often attempted first due to lower perceived invasiveness, and many patients achieve adequate symptom control. The optimal timing of surgical referral remains debated.
2. PERT dosing: fixed versus fat-based individualization. The 2023 AGA Clinical Practice Update recommends a starting dose of ≥40,000 USP units of lipase per meal, but emphasizes subsequent adjustment based on meal fat content and clinical response. Some experts advocate for a more precise “fat-adjusted” dosing (e.g., 2,000–4,000 units per gram of dietary fat), while others argue that patient-reported symptom improvement is sufficient for titration. The absence of head-to-head trials leaves this unresolved.
3. Screening for metabolic bone disease. While DEXA screening is recommended by most experts, the optimal screening interval (e.g., annually, every 2–3 years, or only at diagnosis) and the target population (all CP patients vs. those with specific risk factors) are not standardized. Additionally, the role of vitamin K₂ supplementation remains investigational.
4. Anticoagulation for splanchnic vein thrombosis. There is no randomized evidence to guide anticoagulation in CP-related splanchnic vein thrombosis. Given the frequent spontaneous recanalization (up to 30%) and the risk of variceal bleeding, clinical practice varies widely from observation alone to full-dose anticoagulation. Prospective studies are urgently needed.

## Conclusion

CP is a complex disease with multifactorial origins, often presenting with significant primary complications. Effective management demands specialized clinical and technical expertise from a dedicated, multidisciplinary care team. While early surgical intervention and continuous disease monitoring improve prognosis, key decisions—such as the choice between endoscopic and surgical procedures, optimal timing for intervention, and nutritional optimization strategies—necessitate ongoing collaborative, multimodal discussion.

Looking forward, several promising directions may transform the management of CP-related complications. First, precision medicine approaches—such as genotype-guided risk stratification for patients with hereditary pancreatitis (e.g., PRSS1, SPINK1, CFTR mutations)—could enable earlier identification of individuals at highest risk for specific complications, allowing tailored surveillance and preventive strategies. Second, novel therapeutic targets are emerging from improved understanding of pancreatic fibrogenesis, including anti-fibrotic agents (e.g., connective tissue growth factor inhibitors, hedgehog signaling modulators), gut microbiota-based interventions, and stem cell therapies aimed at preserving or restoring exocrine and endocrine function. Third, the complexity of CP complications increasingly demands integrated, multidisciplinary care models—the “pancreas center” approach—where gastroenterologists, interventional endoscopists, pancreatic surgeons, pain specialists, endocrinologists, dietitians, and radiologists collaborate in a single setting to provide coordinated, patient-centered management. Future research should focus on validating these approaches through prospective multicenter studies and cost-effectiveness analyses.
